# Effect of Climate Change on Mediterranean Winter Ranges of Two Migratory Passerines

**DOI:** 10.1371/journal.pone.0146958

**Published:** 2016-01-13

**Authors:** José L. Tellería, Javier Fernández-López, Guillermo Fandos

**Affiliations:** 1 Departamento de Zoología y Antropología Física, Facultad de Ciencias Biológicas, Universidad Complutense, Madrid, Spain; 2 Real Jardín Botánico de Madrid, Consejo Superior de Investigaciones Científicas, Madrid, Spain; Università degli Studi di Milano-Bicocca, ITALY

## Abstract

We studied the effect of climate change on the distribution of two insectivorous passerines (the meadow pipit *Anthus pratensis* and the chiffchaff *Phylloscopus collybita*) in wintering grounds of the Western Mediterranean basin. In this region, precipitation and temperature can affect the distribution of these birds through direct (thermoregulation costs) or indirect effects (primary productivity). Thus, it can be postulated that projected climate changes in the region will affect the extent and suitability of their wintering grounds. We studied pipit and chiffchaff abundance in several hundred localities along a belt crossing Spain and Morocco and assessed the effects of climate and other geographical and habitat predictors on bird distribution. Multivariate analyses reported a positive effect of temperature on the present distribution of the two species, with an additional effect of precipitation on the meadow pipit. These climate variables were used with Maxent to model the occurrence probabilities of species using ring recoveries as presence data. Abundance and occupancy of the two species in the study localities adjusted to the distribution models, with more birds in sectors of high climate suitability. After validation, these models were used to forecast the distribution of climate suitability according to climate projections for 2050–2070 (temperature increase and precipitation reduction). Results show an expansion of climatically suitable sectors into the highlands by the effect of warming on the two species, and a retreat of the meadow pipit from southern sectors related to rain reduction. The predicted patterns show a mean increase in climate suitability for the two species due to the warming of the large highland expanses typical of the western Mediterranean.

## Introduction

Modern climate change is a main driver of large-scale species distribution, but its actual effects on individual species will result from a variety of context-dependent processes that need to be explicitly studied at the proper scales [1–3]. This involves investigating the way climate change affects the environment requirements of organisms and how they cope with the changes through relocation, phenotypic plasticity or adaptation [4]. Only in this way it is possible to understand the potential effects of changes on species and to design preventive or proactive measures to cope with them realistically [5].

Migratory birds are sensitive to climate change in the different geographical sectors along their migratory journeys [6]. This itinerancy makes it difficult to explore the potential effect of climate change on these species [7]. In the case of European migratory birds, it has been predicted that many trans-Saharan species will reduce and shift their wintering range due to the effect of climate change [8], but little is known about the fate of wintering grounds in the Palaearctic [9]. Some evidence suggests a shift northwards of wintering ranges in some European birds but few studies have investigated the impacts of climate change on wintering areas in the Mediterranean Basin [9]. This is, however, a main wintering ground for many European bird populations [10] because the arrival of autumn rains mitigates the limiting effects of summer drought initiating a period of primary productivity, invertebrate activity and fruit ripening suitable for wintering birds [11].

**Fig 1:**
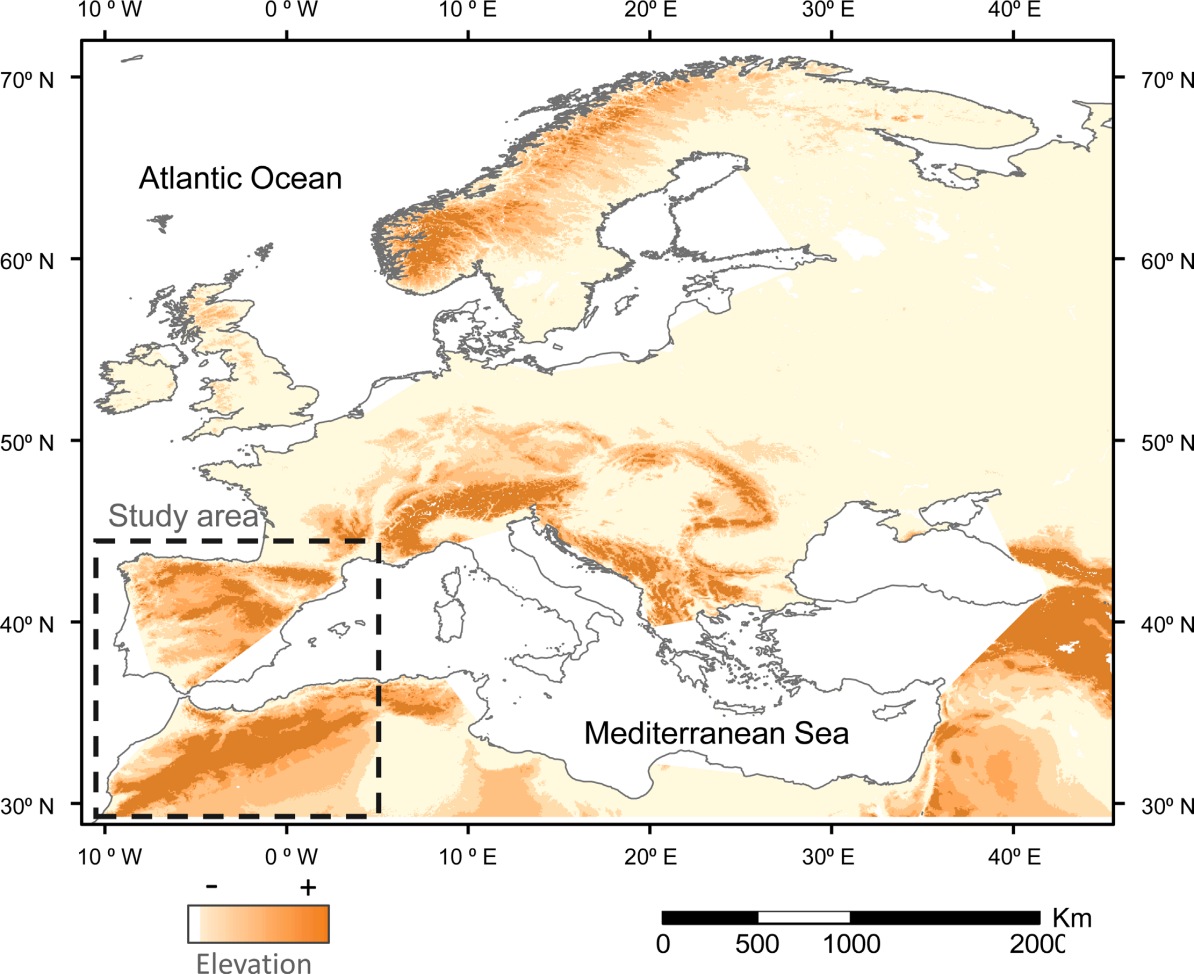
Elevation map of the Western Palaearctic. The study area is located within the stripped square. Increasing dark tones show increasing elevations, with the darkest tone showing the areas over 1,000 m a.s.l.

The Mediterranean basin is located at the southwestern border of the Palaeartic (Fig 1), which is expected to suffer stronger effects in terms of climate change [12,13]. Thus, it is suspected that climate change will influence the extent and suitability of this wintering ground for birds, but no information is available on the potential strength and spatial distribution of changes. These changes have potential conservation implications because can affect the survival or the subsequent reproductive success of migratory birds and thus their populations trends [14–16]. In the current work, we study the potential effect of climate change in two common passerines (the meadow pipit *Anthus pratensis* and the chiffchaff *Phylloscopus collybita*) wintering in the Iberian Peninsula and the Maghreb. More explicitly, we address the following objectives:

### Effects of climate on the present distribution of migratory birds

**Fig 2:**
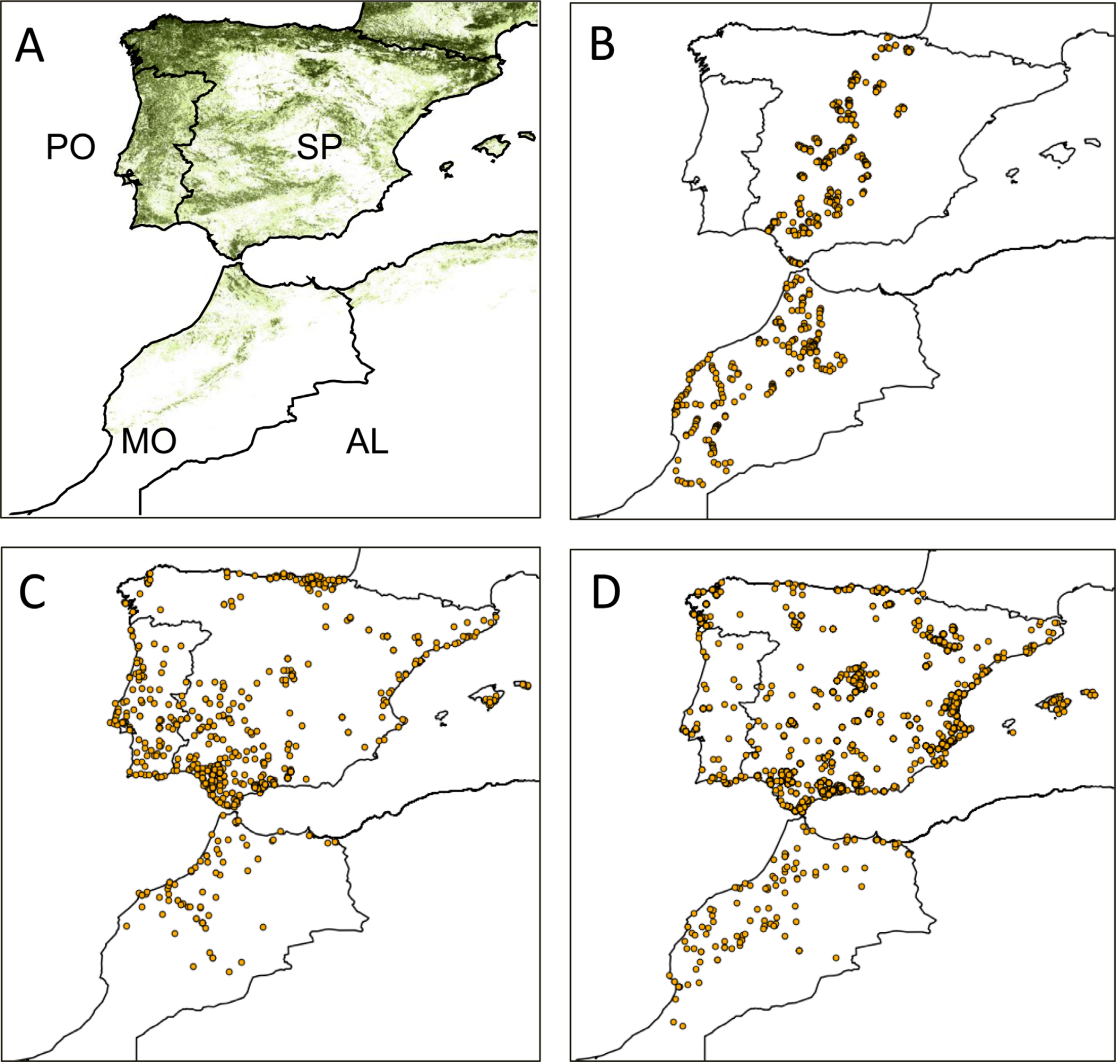
Study area and sampling effort distribution. A. Distribution of tree cover and political division of the study area. AL: Algeria, MO: Morocco, PO: Portugal, SP: Spain. B. Distribution of localities where the occupancy and abundance of species was sampled. C and D. Distribution of winter localities in which ringed meadow pipits and chiffchaffs were recorded.

We assess the effects of climate on the abundance distribution of pipits and chiffchaffs in a set of study localities along a 1500 km-long belt crossing Spain and Morocco (Fig 2). This is a preliminary step to understand the effect of climate change on birds. We assume that climate can affect the distribution of wintering passerines in two main ways. First, food availability (e.g. seeds, fruits, invertebrates) is ultimately derived from plants and the effect of climate on plant productivity (e.g. through precipitation or temperature) influences bird abundance [17,18]. Second, low temperatures increase the energy demands of these small birds, which are then forced to increase food intake to cope with thermoregulatory requirements [19,20]. As a result, it can be postulated that the occupation of warm areas will decrease the need for thermogenesis and thus reduce individual daily energy requirements, enabling more birds to occur at a given level of productivity [21,22]. However, this effect of climate on bird distribution could be affected by the competing effects of other geographical and environmental features [23,24]. For instance, pipits and chiffchaffs arrive at the study area from the north. This seasonal afflux of birds could decrease the number of individuals involved in the colonization of the southern border of the winter range. This is usually related to the positive effect of migration length on mortality and energetic costs [25,26]. Similar effects have already been reported in this wintering ground, where migratory fluxes affect winter distribution of birds [27,28]. In addition, birds are strongly affected by vegetation structure so that the availability of suitable sites will strongly affect their presence in a given area [29]. Thus, given the multiple determinants of bird distribution, we use a multivariate approach to assess the relative contribution of climate vs. other geographical and habitat predictors on the distribution of pipits and chiffchaffs.

### Modelling the present distribution of climate suitability

We use climate variables affecting pipit and chiffchaff abundance to predict the distribution of the most suitable wintering grounds. To do this, we use the spatial distribution of winter ring records provided by the European Union for Bird Ringing (Euring; http://www.euring.org/) to explore the distribution of species. As these recoveries are widely distributed throughout the study area (Fig 2) they can be used to explore the environmental preferences of birds. The models will be constructed with Maxent, a popular machine-learning technique designed to predict the occurrence probabilities of species by combining species presences (latitude and longitude) with the geographical distribution of climate variables [30,31]. The ability of these maps to identify sectors of different climatic suitability will be tested with the distribution of pipits and chiffchaffs in the study localities (Fig 2). If the actual occupancy and abundance [32] of birds agree with the occurrence probability maps, we will use the models to forecast the changes in the extent and suitability of wintering areas according to predictions of climate change. This approach assumes a "space-for-time" design, which attempts to predict temporal changes from present spatial correlates between species distribution and climate variables [33].

### Effects of climate change on the winter distribution of migratory birds

Finally, we use the validated distribution models to forecast the suitability of wintering grounds according to climate projections. The effect of climate change on breeding bird distribution has already been studied in the western Mediterranean [34,35], but little is known about the way climate change will affect winter bird ranges. It has been predicted, for instance, that warming will improve the suitability of colder sectors, such as mountain and highlands [36]. Climate predictions also suggest a reduction in precipitation in some southern areas that could reduce primary productivity [37,38] and the concomitant suitability of present wintering grounds for some species [39,40]. Thus, climate changes will likely be affected by latitudinal and altitudinal gradients producing a patchy distribution of the future climate trends. This study attempts to map the location and extent of these changes in climate suitability of the Iberian Peninsula and the Maghreb for the two study species.

## Materials and Methods

### The model species

The meadow pipit (mean body mass 18.4 g; [41]) feeds on invertebrates in meadows and grasses of open habitat patches [42]. The chiffchaff (7.8 g) feeds on insects within a broader set of substrata and occurs in tree- and shrub-covered habitats [43]. These passerines were selected as model species for two reasons. First, they do not breed in the study area (the meadow pipit) or mainly breed in some areas of the northern Iberian Peninsula (the chiffchaff; [44]). Thus, most if not all birds wintering in the study area are migratory individuals [45–47]. This trait is interesting from a methodological point of view because it will attenuate the confounding effects of local conspecifics on the abundance patterning of migratory individuals [48]. In this way, predictions based on ring recoveries in wintering grounds can be validated by the abundance distribution of the same migratory populations [49]. Second, pipits and chiffchaffs are small insectivorous passerines that occur in a broad range of habitats [42,43]. Small body size makes birds more sensitive to temperature [20] and a diet based on insects makes them potentially ubiquitous since these invertebrates are available in a wide range of habitats [50]. This implicitly assumes the potential of these species to track climate changes across the entire study area.

### Study area

**Fig 3:**
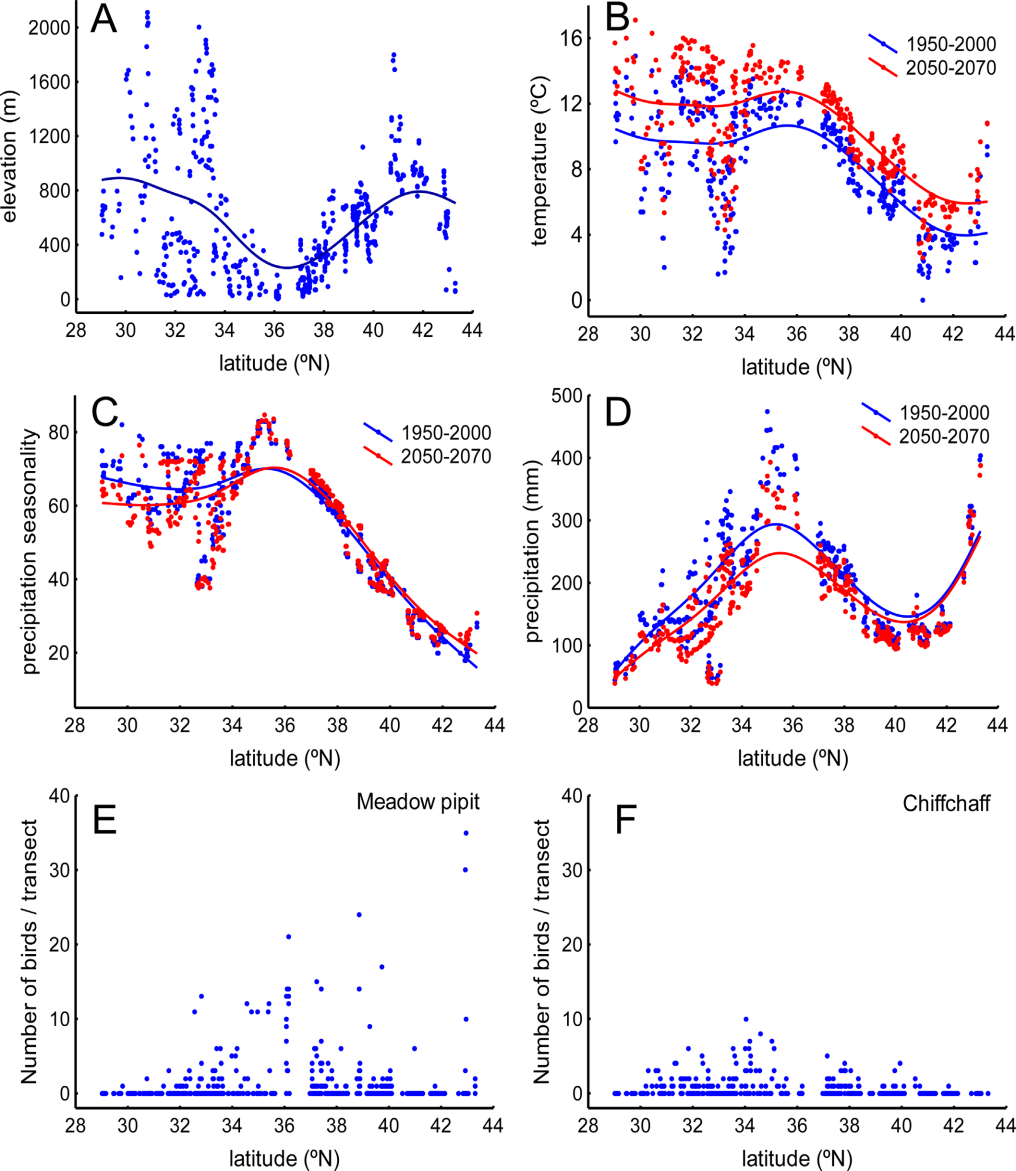
Elevation, climate and bird abundance distribution along the latitudinal gradient defined by the sampling localities in Fig 1B. A) Elevation, B) temperature, C) precipitation seasonality, D) precipitation. Climate variables are averages provided by the WorldClim-Global Climate Data facility (http://www.worldclim.org) for the 1950–2000 and 2050–2070 periods (see text). The abundances along the gradient of the meadow pipit (E) and the chiffchaff (F) are also reported.

The study area is located between 28° and 44°N at the southwestern border of the Palaearctic, covering an area of 1,700,000 km^2^ (Fig 1). It is a transitional, Mediterranean region located between the moist woodlands and meadows of the northern and western half of Iberia and the dry expanses of the Sahara (Fig 2). The area is covered by extensive bare lands, grasslands and farmlands (e.g., cereal fields, olive groves) interspersed with Mediterranean scrublands (*Pistacia lenticus*, *Olea europaea*, etc.) and oak (*Quercus ilex*, *Quercus suber*, etc.) and conifer (*Pinus halepensis*, *Pinus pinaster*, etc.) woodlands. The most outstanding trends along this latitudinal gradient are the sharp changes in elevation and the concomitant changes in climate conditions (Fig 3).

### Effects of climate on the present distribution of birds

#### Field sampling

Field work was carried out from 2006 to 2013 to assess meadow pipit and chiffchaff abundance in 556 localities ranging from 7 to 2110 m a.s.l. along a 1500 km belt crossing the Iberian Peninsula and the Maghreb (Fig 2). Permissions were not required because we counted birds in free-access lands. At these points we recorded all individuals seen or heard at either side of 500 m-long line transects irrespective of the perpendicular distance at which each individual was detected. The number of individuals recorded per line transect is a common index of bird abundance in extensive bird counts [51]. In each transect, we measured some vegetation traits in two 25-m radius circles separated by 200-m intervals. In each circle, we visually estimated grass cover and shrub and tree layers (shrub under 0.5 m, between 0.5–2 m and >2 min height). We also recorded the number of tree and shrub species within the circles. The scores of the two sampling circles were averaged to characterize each line transect (S1 Table). We conducted a principal components analysis to reduce tree and shrub cover and richness to just one score. We retained a single principal component (eigenvalue: 2.80), which explained 55.94% of variance and was interpreted as a gradient of increasing vegetation cover (factor loadings for vegetation cover under 0.5m height: -0.01; cover 0.5–2 m height; 0.84; cover >2 m height: 0.77; shrub species: 0.84 and tree species: 0.90). Factor scores of each locality within the component were used as an index of shrub and tree development (woodland). The location of locality (latitude and longitude in decimal degrees) was recorded with GPS devices.

#### Climate

We assessed climate conditions of the study sites by using mean scores provided by Worldclim 1.4 for the 1950–2000 period [52]. Since we propose that small birds avoid cold areas to prevent excessive thermoregulatory requirements, and precipitation may affect the productivity of Mediterranean habitats after summer drought (see Introduction), we selected Mean Temperature of Coldest Quarter (bio11) and Precipitation of Coldest Quarter of the year (bio19) as the two main climate correlates of bird distribution. In addition, we included Precipitation Seasonality (bio15) as a surrogate of the rain-mediated productive pulses typical of many dry habitats that track migratory birds [53,54]. Finally, to test if the present spatial patterning of climate variables is similar to that provided by Worldclim for the second half of the twentieth century, we requested from World Weather (http://en.tutiempo.net/) the available climate data (years 2012–2013) in 25 meteorological stations located along the study area (from Avilés 43.55°N, 6.03°W to Sidi Ifini 29.36°N, 10.18°W). Despite strong differences in sample size affecting the means (50 vs. 2 years), climate variables depicted similar trends (bio 11, r: 0.93; bio15:0.69 and bio19:0.70, p<0.001 in all cases) supporting the usefulness of climate data used in this paper to explore the features affecting the present spatial distribution of birds.

#### Analyses

We used meadow pipit and chiffchaff abundance distribution as response variables and climate (temperature, precipitation, precipitation seasonality) and vegetation structure (grass and woodland cover) as predictor variables. The effect of latitude (and the potential effect of spatial autocorrelation) on bird abundance was also included by means of the line transects’ coordinates, latitude, longitude and latitude x longitude [55]. To deal with the large number of zeros in transects (Fig 2) we used the hurdle count model approach provided by pscl-R [56]. These models are two-component models with a hurdle component that models the zero counts and a truncated count component for positive counts [57]. The count model is normally a truncated Poisson or negative binomial regression (with log link). The hurdle model is a bionomial logit regression or a censored count distribution model [56]. We used the Akaike's Information Criterion [58] to select the best model and variable combination with lmtest-R [59].

### Modelling the present distribution of climate suitability

#### Ring recovery distribution modelling

We used the localities where ringed birds were recorded in December, January and February (374 for the meadow pipit and 516 for the chiffchaff; duplicate records in the same localities were removed in each replicate) as presence data (Fig 2). These records result from birds ringed in breeding grounds or during the migratory displacements that have been recovered in the study region from 1960 to 2010. Most of meadow pipits ringed in breeding grounds (n = 63) arrived from the United Kingdom (55%) and Germany (17%) while most of the chiffchaffs (n = 81) arrived from Germany (35%), Belgium (26%) and France (17%).The geographical location of ring recovery localities (latitude and longitude) was used to run Maxent combined with the climate variables (temperature, precipitation, precipitation seasonality; see above). Since ring recovery distribution can be affected by human activity (e.g. more records are gathered in the most populated areas) [10], the layer of human footprint (an index of population density, land transformation and road density) [60] was used as a bias grid to distribute 10,000 background points with a likelihood of presence proportional to the human footprint index. Maxent (log output; regularization multiplier b = 1; autofeatures; convergence threshold = 0.00001) was run in 10 replicates with 75% of the presences as training data and the rest as test data for internal verification. The Area Under the Curve (AUC) provided by the receiver operating characteristic curves was used to assess the congruence between observed and detected records in the test data reserved for verification in Maxent [30].

#### External validation

We used the data provided by line transects to test the ability of Maxent models to predict bird distribution. To do this, we divided the occurrence probability resulting from Maxent into three similar interval sectors between the min-max intervals. The resulting ranges were used to define maps of low, medium and high climate suitability for each species. The line transects were distributed within these sectors to test with χ^2^ or ANOVA analyses if the number of localities at which the species are found to occur (occupancy) [32] and mean abundance increased from low to high climate-suitable sectors [49]. In addition, since the distribution models were launched to predict climate suitability, the effect of habitat structure on bird distribution was controlled by selecting the habitats mainly used by pipits (grasslands, farmlands, wooded pasturelands; n = 375) and chiffchaffs (wooded pasturelands, farmlands, scrublands, woodlands; n = 320) according to current information on winter habitat preferences [45,47]. In this way, we tried to avoid the absences resulting from the unsuitability of some habitats.

### Effects of climate change on bird distribution

After validation, the Maxent models were used to project the changes in species distribution under RCP2.6 and RCP8.5 scenarios for 2050 and 2070 (AR5 report) [61] provided by the WorldClim-Global Climate Data facility (http://www.worldclim.org/). Scenario RCP2.6 is representative of mitigation policy aiming to limit the increase of global mean temperature to 2°C. Scenario RCP8.5 does not include any mitigation target [62]. We used two independent global circulation models (MIROC5 and CCSM4) suitable for the study area to predict the changes. To obtain a straightforward view of the species distribution in the future, we also averaged all predicted trends for scenarios, models and time periods (2050 and 2070).

Present occurrence probabilities were subtracted from future occurrence probabilities to map the distribution of increasing and decreasing climate suitability sectors. To explore the spatial distribution of climate and climate suitability changes for pipits and chiffchaffs, we distributed 500 random points over the Iberian Peninsula and Morocco (there were actually 493 because seven points were discarded for lack of data). In each point, the reported changes in climate suitability and climate variables were used as response variables and latitude, longitude, latitude x longitude, altitude and altitude^2^ (to detect hump shaped distributions along the elevation gradient) were used as predictors. Present and future scores were compared by repeated measures ANOVA and the spatial trends of changes by GLM analyses. All cartographic data were managed with QGIS 2.8 and GRASS GIS in 10 x 10 km squares [63]. Statistical analyses were carried out with Statistica 8.1 (StatSoft, USA).

## Results

### Effects of climate on the present distribution of migratory birds

A negative binomial distribution for the count model and a logistic regression for the binomial model was the best zero hurdle model for the two species (Table 1). The meadow pipit occurred more frequently in warm and rainy areas covered by grasses with low woodland cover, and the chiffchaff was more frequent in southern, warm sites covered by woodlands. The best count model was a zero altered negative binomial regression with log link (Table 1). According to this model, the meadow pipit was more abundant in northern, rainy sites covered by grasses and the chiffchaff in warm sites covered by grasses. The two methodological approaches support an effect of climate variables on the species distribution, with more frequent and abundant meadow pipits in warm and rainy sectors and more frequent and abundant chiffchaffs in warmer areas.

**Table 1:** Results of hurdle count models to explain meadow pipit and chiffchaff distribution in wintering grounds of the Iberian Peninsula and the Maghreb.

	Zero hurdle model				Count model			
	Meadow pipit		Chiffchaff		Meadow pipit		Chiffchaff	
	Estimate	P	Estimate	P	Estimate	P	Estimate	P
**Intercept**	-18.15	<0.001	7.47	0.206	-16.15	0.004	-13.98	<0.001
**Lat.**	-	-	-0.49	<0.001	0.20	0.013	-	-
**Lon.**	1.17	<0.001	2.46	0.003	0.54	0.009	-	-
**Lat. x Lon.**	-0.04	<0.001	-0.07	0.003	-	-	-	-
**Temperature**	3.54	<0.001	3.95	<0.001	-	-	6.78	<0.001
**Prec. season.**	-	-	-	-	-2.41	0.280	-	-
**Precipitation**	3.37	<0.001	-	-	4.25	<0.001	-	-
**Grass**	0.92	<0.001	-	.	0.61	0.041	0.87	<0.001
**Woodland**	-1.49	<0.001	0.92	<0.001	-	-	-	-

Zero hurdle models show the features related to the occupancy of species and count models the features affecting abundance.

### Modelling the present climate suitability

**Fig 4:**
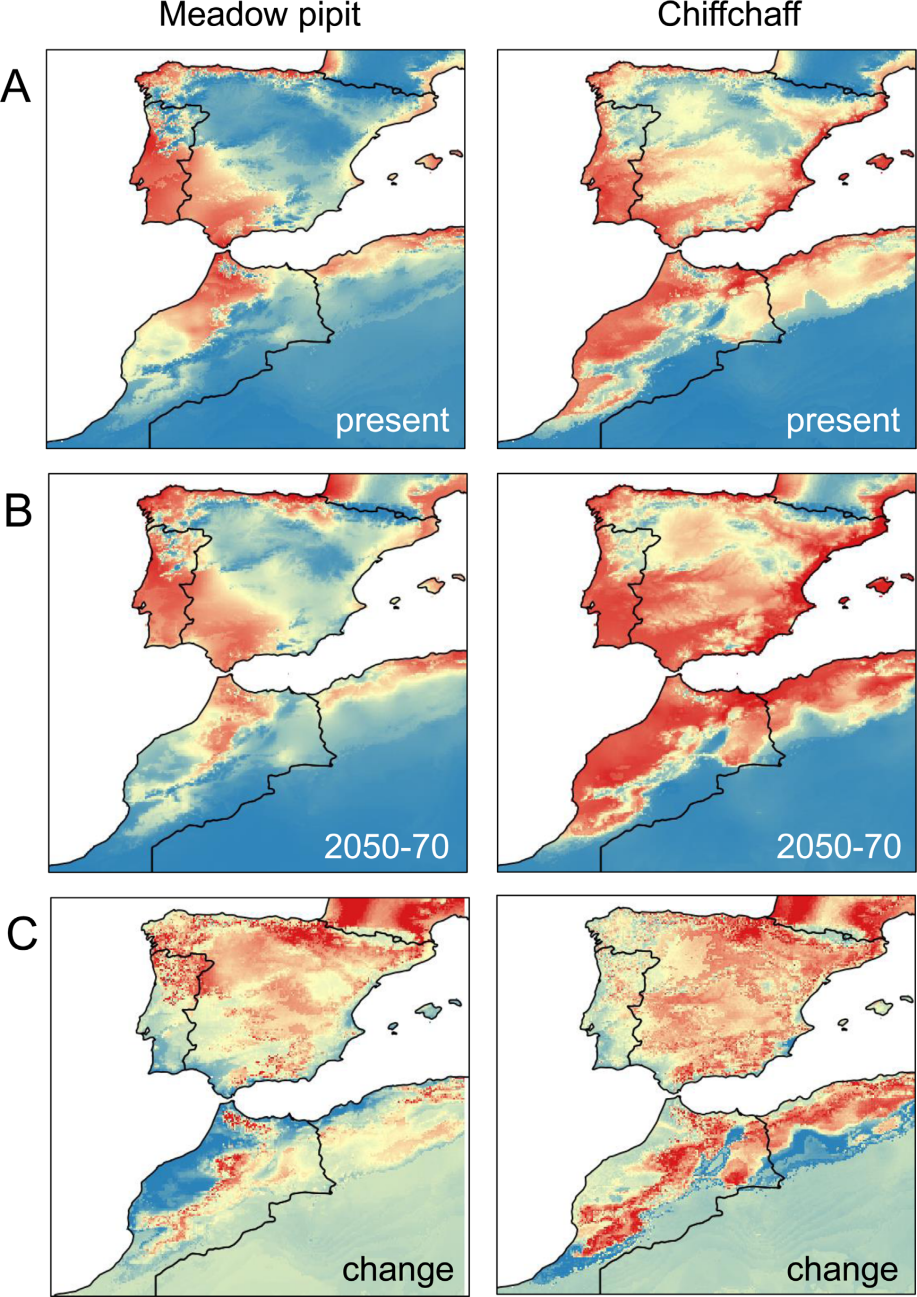
Trends of climate suitability. A. Distribution of high (red), medium (yellow) and low (blue) climate suitability sectors for the meadow pipit and the chiffchaff according to similar occurrence probability intervals. B. Mean predicted distributions for the period 2050–2070. C. Change in climate suitability from present to 2050–2070. Blue color represents decrease and yellow to red represent increasing rates of climate suitability.

Winter temperatures and precipitations were the most important variables in modelling the occurrence probabilities of the meadow pipit and the chiffchaff with Maxent (Table 2). In the two species, the most suitable areas were located on coastlines and in lowlands of the study region (Fig 4). The occupancy and abundance distribution of the two species fitted well to the distribution of low, medium and high climate suitability sectors resulting from ring recovery occurrences (Table 3). As predicted, the main difference between the all habitat vs. suitable habitats approaches was related to increased occupancy and abundance scores in suitable habitats. This supports the ability of occurrence probabilities provided by Maxent to predict the distribution of pipits and chiffchaffs in the Iberian Peninsula and the Maghreb.

**Table 2:** Estimates of the effect of the climate variables on Maxent models.

Species	Variable	Percent contribution	Permutation importance
**Meadow pipit**	Temperature (bio11)	42.4	65.4
	Precipitation seasonality (bio15)	3.8	5.3
	Precipitation (bio19)	53.8	65.4
	AUC ± SD	0.877±0.018	
**Chiffchaff**	Temperature (bio11)	48.2	29.1
	Precipitation seasonality (bio15)	12.2	9.6
	Precipitation (bio19)	39.6	61.3
	AUC ± SD	0.836±0.017	

Percent contribution indicates the change in regularized gain by adding the corresponding variable. Permutation importance represents, for each variable in turn, the resulting drop in training AUC when the values of that variable on training presence and background data are randomly permuted. They are normalized to show percentages. Values are averages over 10 replicate runs.

**Table 3:** Distribution of the species occupancy and abundance reported by field sampling among the three sectors of climate suitability defined by the occurrence probabilities resulting from species distribution models.

	Species distribution model		Field sampling	
	Occurrence probability interval	Climate suitability	Occupancy (presences/absences)	Abundance (no birds/transect ± SE)
**Meadow pipit**	0.01–0.31	Low	0.14 (43/262)	0.47 ± 0.18
**(all habitats)**	0.32–0.58	Medium	0.26 (37/108)	1.11 ± 0.26
	0.59–0.84	High	0.34 (27/52)	2.73 ± 0.33
			χ^2^ = 32.24, P<0.001	F_2,553_ = 33.84, P<0.001
**Meadow pipit**	0.01–0.25	Low	0.18 (26/115)	0.62 ± 0.33
**(suitable habitats)**	0.25–0.58	Medium	0.31 (42/90)	1.31 ± 0.34,
	0.58–0.84	High	0.54 (55/47)	2.87 ± 0.38,
			χ^2^ = 33.90, P<0.001	F_2,372_ = 10.22, P<0.001
**Chiffchaff**	0.01–0.24	Low	0.11 (20/169)	0.15 ± 0.08
**(all habitats)**	0.24–0.48	Medium	0.21 (42/201)	0.37 ± 0.07
	0.48–0.74	High	0.42 (48/66)	1.15 ± 0.09
			χ^2^ = 46.15, P<0.001	F_2,553_ = 36.91, P<0.001
**Chiffchaff**	0.01–0.26	Low	0.11 (5/41)	0.13 ± 0.20
**(suitable habitats)**	0.26–0.49	Medium	0.25 (41/123)	0.41 ± 0.11
	0.50–0.74	High	0.70 (77/33)	1.76 ± 0.13
			χ^2^ = 73.60, P<0.001	F_2,317_ = 53.35, P<0.001

The results of χ^2^ and ANOVA analyses to test for differences in species occupancy and abundance are shown.

### Effects of climate change on bird distribution

**Fig 5:**
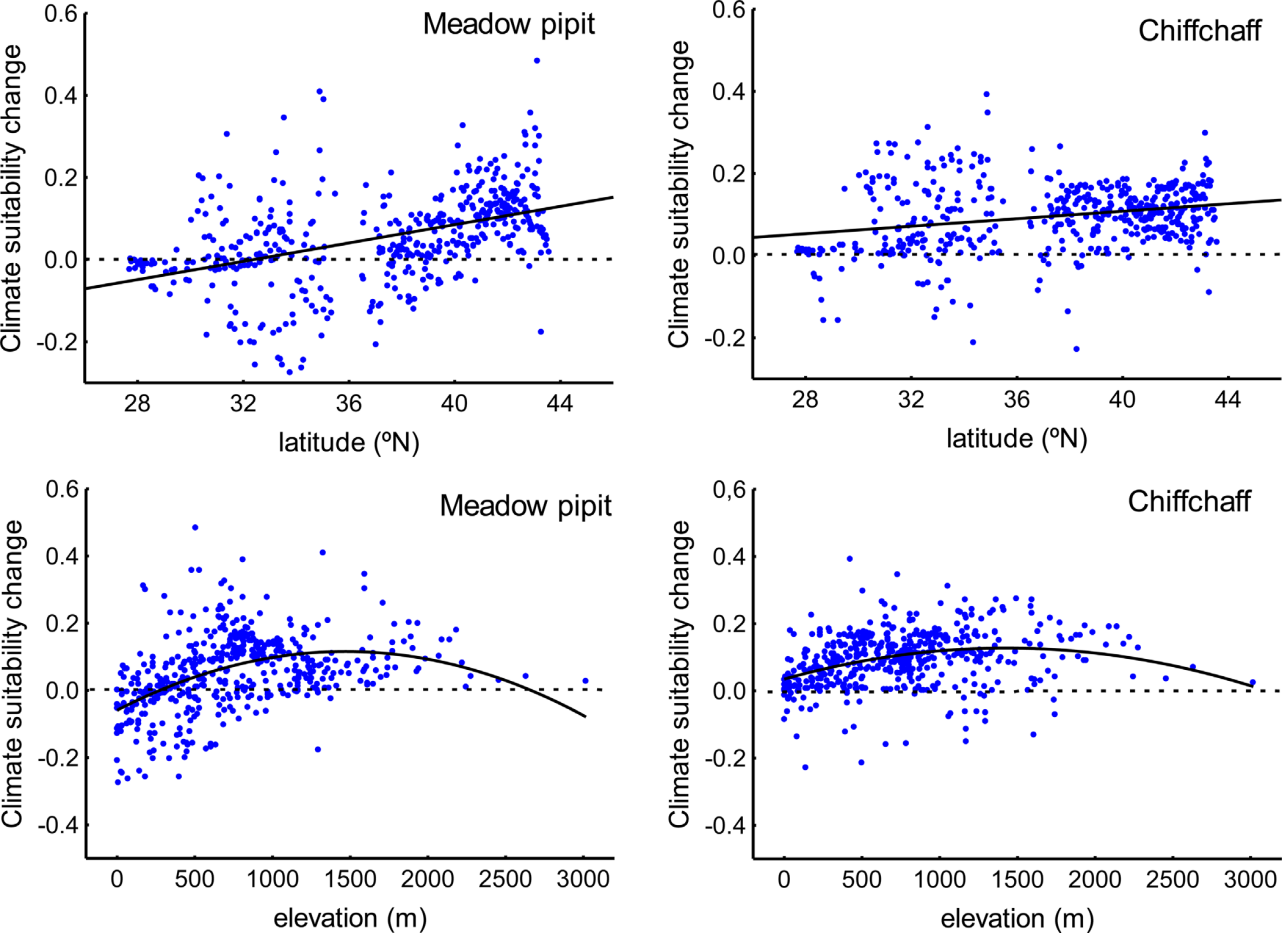
Latitudinal and altitudinal change in the climate suitability for wintering meadow pipits and chiffchaffs across the study region. These results have been reported by 493 random distributed sampling points across the Iberian Peninsula and Morocco (see text).

According to predictions for the period 2050–2070, winter mean temperature will increase (mean ± SE, 1950–2000: 7.47 ± 0.16°C; 2050–2070: 9.28 ± 0.16°C; repeated measures ANOVA F_1,492_ = 7,240.27, P<0.001) and winter mean precipitation (177.732 ± 5.057 mm,161.02 ± 4.73 mm; F_1,492_ = 518.22, P<0.001) and winter precipitation seasonality (48.34 ± 0.82 mm,42.73±0.64 mm; F_1,492_ = 513.22, P<0.001) will decrease in the Iberian Peninsula and the Maghreb. These trends will be followed by a predicted increase in winter mean climate suitability for the meadow pipit (mean ± SE, 1950–2000: 0.28 ± 0.01; 2050–2070: 0.33 ± 0.01; repeated measures ANOVA F_1,492_ = 114.21, P<0.001) and the chiffchaff (0.36 ± 0.01; 0.46±0.0.01 mm; F_1,492_ = 663.53, P<0.009). The changes will not be distributed evenly across the region (Fig 4). For instance, the increase in temperature and the loss of precipitation (and precipitation seasonality) will be reduced in northern sites (Table 4). Elevation will also affect climate change suggesting a hump-shaped relationship with temperature increase and precipitation reduction (the changes will be reduced at mean altitudes; Table 4). As a result, climate suitability for the meadow pipit and the chiffchaff will shift from lowlands to highlands and from southern to northern areas (Table 4; Fig 5).

**Table 4:** Mean trends of climate variables and occurrence probabilities (o.p.) of birds between 1950–2000 and 2050–2070 in the study area.

	/Trends		GLM analysis							
	mean ± SE	Min / max	Latitude (ß)	Longitude (ß)	Lat. x Long. (ß)	Elevation (ß)	Elevation^2^ (ß)	F_5,486_	P	R^2^
**Temperature (°C)**	+2.02 ± 0.02	—1.20 / +5.20	-0.21	-	0.73	0.45	-0.30	27.84	<0.001	0.22
**Precipitation seasonality**	-5.61 ± 0.25	-3.38 / +5.50	0.42	1.56	-1.39	-	-	351.10	<0.001	0.78
**Precipitation (mm)**	-1.67 ± 0.07	-10.98 / +2.60	0.75	-2.12	2.13	0.48	-0.53	24.22	<0.001	0.20
**Meadow pipit (c.s.)**	+0.05 ± 0.01	-0.27 / +0.49	0.89	-1.37	1.14	1.04	-0.57	85.03	<0.001	0.47
**Chiffchaff (c.s.)**	+0.10 ± 0.01	-0.23 / +0.39	0.42	**-**	0.66	0.66	-0.37	23.07	<0.001	0.19

The results of GLM analyses on the effects of geographical location and elevation on changes are shown. Beta (ß) figures indicate the magnitude and sign of the partial relationships of predictor variables. Betas of non-significant effects are not shown.

## Discussion

### Effects of climate on the present distribution of migratory birds

Species distribution is shaped by features acting at different scales [64]. In the case of migratory birds, distribution results from large-scale geographical and climate processes affecting the historical configuration of migratory routes [65] and the local arrangement of individuals according to their particular habitat preferences [29]. Multi-scale approaches are thus required to detect the contribution of climate versus other features on the distribution of migratory birds [23,66]. This has been the aim of the present study in which the distribution of wintering pipits and chiffchaffs has been related to large scale (geography and climate) and local (vegetation cover) predictor variables (Table 1).

The effect of latitude on bird distribution provided inconclusive results. The meadow pipit was more abundant in the northernmost sectors supporting the predicted effect of the distance to breeding grounds on winter distribution. However, the occupancy of the chiffchaff showed an opposite pattern, perhaps because in this species many individuals winter in the Sahel [43]. Thus, the study area is not the actual southern border of its wintering area. After controlling for the geographical effects, the results supported habitat structure and climate as key players in bird distribution. Woodland cover was related to the occurrence of the chiffchaff (positive) and the meadow pipit (negative). In both cases, abundance was positively related to grass cover. These results are congruent with the habitat preferences of the species since the meadow pipit prefers grass patches in open habitats and the chiffchaff feeds on a broad variety of substrata (included tall grasses) in shrub- and tree-covered habitats [42,43].

Climate variables were related to bird distribution supporting the presence of more pipits and chiffchaffs in warmer sectors (Table 1). Preferences for warmer areas could be explained by the predicted direct effect of low temperatures on thermoregulation costs (see Introduction). In an area dominated by cold mountains and highlands (Fig 1), where most sectors show mean winter temperatures (mean ± SE: 9.36°C ± 0.18; min-max: -2.2 to 16.7°C; n = 493; see methods) under the usual thermoneutral zone of passerines [67], birds may be forced to increase metabolism [68] or to leave colder sectors [36,69]. In addition to this effect of thermal landscape on bird distribution, meadow pipits were positively related to precipitation. Autumn and winter rains mitigate the effect of summer drought in the Mediterranean and favour primary productivity and food availability for wintering birds [11, 39,40]. This effect could be magnified by the use of ground by the meadow pipits as feeding substratum [42]. In the Mediterranean region, grass growth and the superficial activity of ground invertebrates are strongly related to the moisture produced by autumn and winter rains on dry ground layers resulting from summer drought [38,70,71]. In this way, more precipitation will result in more suitable food patches for this species [48]. Thus, it can be concluded that rain-mediated productivity and thermal landscapes can provide a causal explanation of climate features affecting the distribution of these small passerines in the study area.

### Modelling the distribution of climate suitability

Temperature and precipitation were the best contributors to the models such that the resulting maps depicted similar distribution patterns in both species (Fig 4). The sectors with high occurrence probabilities were distributed in lowlands and low occurrence areas were restricted to highlands and inland deserts. There were, however, some differences. While the meadow pipit was mostly related to western lowlands where Atlantic rains drain, the chiffchaff showed a broader distribution. Interestingly, the models fitted well to the present distribution of the species, with higher occupancy and abundance in sectors where the Maxent models suggest high climate suitability (Table 3). This supports the usefulness of models resulting from ring recoveries and suitable climate variables to predict the geographical distribution of wintering in these birds [54].

### Effects of climate change on bird distribution

According to IPCC predictions [72], mean temperature will increase and precipitation will decrease in the study area over the next few decades. These changes will not be spatially homogeneous in the study area. Some southern sectors will be more affected by changes (Fig 3) and, within this latitudinal trend, temperature increases will grow and precipitation loss will decline in the highlands (Table 4). This change has been reported in other studies of the effect of elevation on climate change [73,74]. As a result, and according to the reported effects of winter temperature and precipitation on the meadow pipit and chiffchaff, the models predict two main trends in climate suitability. First, mean climate suitability will improve for the two species in the Iberian Peninsula and the western Maghreb. This change seems to be related to the warming of highlands in a region where 80% of the land is over 300 m and 60% over 600 m a.s.l. (Fig 1). In this way, the improvement of winter conditions produced by warming of elevated areas, similar to that reported in northern areas, will provide small birds the possibility to cope with winter conditions more successfully [36]. Second, despite the overall improvement of winter conditions, some sectors will be more vulnerable to the damaging effects of climate change in some species. This is true for the warmer lowlands of the south where the predicted decrease in precipitation will reduce the climate suitability for the meadow pipit, a species linked to moist areas (see above). The fate of water resources is a key concern in the drier sectors of the Iberian Peninsula and the Maghreb [12,13,37].

### Prospects

This study suggests five main aspects for consideration in the assessment of the weaknesses and strengths of the resulting patterns. First, it is important to consider the representativeness of the two model species. They are insectivorous and ubiquitous birds similar to many other European partial-migratory passerines [10]. However, the alleged ability of pipits and chiffchaffs to track climate change over the study region may not apply to stenotopic species or birds forced to track resources with patchy distribution (e.g. water birds, frugivorous species). Second, it is important to state that these results refer to a scenario in which other features (e.g. vegetation cover and composition, agricultural practices, etc.) will also vary as a response to climate change [75]. As a result, climate change could promote synergetic interactions within and among species, evolutionary and ecological processes and human impact that are difficult to predict [5,33]. Third, temperature and precipitation appear to be two main drivers of bird distribution because changes in these parameters affect the energy available for birds (see Introduction). Since energy availability has been reported to be a global driver of bird distribution, changes in these variables are probably suitable indicators of changes in other species and regions [76–77]. Finally, the results highlight the main role of elevation in shaping the retreat of low latitudinal margins of Mediterranean winter ranges of birds reported in this paper. This role of elevation will probably apply in other highlands of the Mediterranean basin (Fig 1) and the planet where winter conditions for birds can benefit from warming [67,70,78]. This main role of highlands in reshuffling the wintering ranges of migratory birds in the Mediterranean basin suggests two additional observations: a) Mediterranean highlands are usually reported as refuges for species retreating northwards due to the effect of postglacial warming [79]. The results in this paper suggest a similar buffer effect on wintering ranges of migratory birds. The huge expanses of Mediterranean highlands (Fig 1), usually avoided by many birds in winter [39–40], would progressively increase climate suitability as warming progresses. b) However, this projected climate improvement of highlands will not be sufficient to predict an increase in migratory populations in this wintering region since warming in northern areas may reduce migratory journeys to the south [3]. Alternatively, it has been postulated that warming of Mediterranean highlands could exert a positive influence on the size of local bird populations favouring winter residency and enhancing winter survival [28].

## Supporting Information

S1 Table Distribution of bird numbers and habitat structure in sampling points along the Iberian Peninsula and the Maghreb. (CSV)

## References

[1] Parmesan C. Ecological and evolutionary responses to recent climate change. Annu Rev Ecol Evol Syst. 2006; 637–669.

[2] Stenseth NC, Mysterud A, Ottersen G, Hurrell JW, Chan KS, Lima M. Ecological effects of climate fluctuations. Science. 2002; 297:1292–1296. 12193777 10.1126/science.1071281

[3] Walther GR, Post E, Convey P, Menzel A, Parmesan C, Bairlein F et al Ecological responses to recent climate change. Nature. 2002; 416: 389–395 11919621 10.1038/416389a

[4] Heller NE, Zavaleta ES. Biodiversity management in the face of climate change: a review of 22 years of recommendations. Biol Conserv. 2009; 142: 14–32.

[5] Moritz C, Agudo R. The future of species under climate change: resilience or decline? Science. 2013; 341: 504–508. 10.1126/science.1237190 23908228

[6] Knudsen E, Lindén A, Both C, Jonzén N, Pulido F et al Challenging claims in the study of migratory birds and climate change. Biol Rev. 2011; 86: 928–946. 10.1111/j.1469-185X.2011.00179.x 21489123

[7] Sanderson FJ, Donald PF, Pain DJ, Burfield IJ, Van Bommel FP. Long-term population declines in Afro-Palearctic migrant birds. Biol Conserv. 2006; 131: 93–105.

[8] Barbet-Massin M, Walther BA, Thuiller W, Rahbek C, Jiguet J. Potential impacts of climate change on the winter distribution of Afro-Palaearctic migrant passerines. Biol Lett. 2009; 5: 248–251. 10.1098/rsbl.2008.0715 19324660 PMC2665829

[9] Pautasso M. Observed impacts of climate change on terrestrial birds in Europe: an overview. Ital J Zool. 2012; 79: 296–314.

[10] Busse P. European passerine migration system–what is known and what is lacking. Ring. 2001; 23: 3–36.

[11] Nahal I. The Mediterranean climate from a biological viewpoint In: Di Castri F, Goodall DW, Specht RL, Ecosystems of the world 11: Mediterranean-type shrublands. Amsterdam: Elsevier; 1981 pp. 63–86.

[12] Giorgi F, Lionello P. Climate change projections for the Mediterranean region. Glob Planet Chang. 2008; 63: 90–104.

[13] Schilling J, Freier KP, Hertig E, Scheffran J. Climate change, vulnerability and adaptation in North Africa with focus on Morocco. Agr Ecosyst Environ. 2012; 156: 12–26.

[14] Rappole JH, King DI, Diez J. Winter vs. breeding-habitat limitation for an endangered avian migrant. Ecol Appl. 2003; 13: 735–742.

[15] Norris DR, Marra PP, Kyser TK, Sherry TW, Ratcliffe LM. Tropical winter habitat limits reproductive success on the temperate breeding grounds in a migratory bird. P Roy Soc Lond B Bio. 2004; 271: 59–64. 10.1098/rspb.2003.2569 PMC1691559 15002772

[16] Kirby JS, Stattersfield AJ, Butchart SH, Evans MI, Grimmett RF. Key conservation issues for migratory land-and waterbird species on the world’s major flyways. Bird Conserv Int. 2008; 18: S49–S73.

[17] Lennon JJ, Greenwood JJD, Turner JRG. Bird diversity and environmental gradients in Britain: a test of the species–energy hypothesis. J Anim Ecol. 2000; 69: 581–598.

[18] Evans KL, James NA, Gaston KJ. Abundance, species richness and energy availability in the North American avifauna. Global Ecol Biogeogr. 2006; 15: 372–385.

[19] Calder WA, King JR. Thermal and caloric relations of birds In: Farner DS, King J. Avian Biology, vol. 14 London: Academic Press; 1974 Pp. 259–413.

[20] Schmidt-Nielsen K. Scaling: why is animal size so important? Cambridge: Cambridge University Press; 1984.

[21] Wright DH. Species–energy theory: an extension of species–area theory. Oikos. 1983; 41: 496–506.

[22] Meehan TD, Jetz W, Brown JH. Energetic determinants of abundance in winter land bird communities. Ecol Lett. 2004; 7: 532–537.

[23] Seoane J, Bustamante J, Díaz-Delgado R. Competing roles for landscape, vegetation, topography and climate in predictive models of bird distribution. Ecol Model. 2004; 171: 209–222.

[24] Melles SJ, Fortin MJ, Lindsay K, Badzinski D. Expanding northward: influence of climate change, forest connectivity, and population processes on a threatened species' range shift. Global Change Biol. 2011; 17: 17–31.

[25] Wikelski M, Tarlow EM, Raim A, Diehl RH, Larkin, RP, Visser GH. Costs of migration in free-flying songbirds. Nature. 2003; 423: 704 12802324 10.1038/423704a

[26] Newton I. The migration ecology of birds London: Academic Press; 2008.

[27] Tellería JL, Ramírez A, Galarza A, Carbonell R, Pérez-Tris J, Santos T. Do migratory pathways affect regional abundance of wintering birds? A test in Northern Spain. J Biogeogr. 2009; 36: 220–229.

[28] Cano LS, Pacheco C, Refoyo P, Tellería JL. Geographical and environmental factors affecting the distribution of wintering black storks *Ciconia nigra* in the Iberian Peninsula. J Avian Biol. 2014; 45: 514–521.

[29] Hutto RL. Habitat selection by non breeding, migratory land birds In: Cody M, Habitat selection in birds. New York: Academic Press; 1985 pp 455–476.

[30] Phillips SJ, Anderson RP, Schapire RP. Maximum entropy modeling of species geographic distributions. Ecol Mode. 2006; 190: 231–259.

[31] Elith J, Phillips SJ, Hastie T, Dudík M, Chee Y E, Yates CJ. A statistical explanation of MaxEnt for ecologists. Diver Distrib. 2011; 17: 43–57.

[32] Gaston KJ, Blackburn TM, Greenwood JJ, Gregory RD, Quinn RM, Lawton JH. Abundance–occupancy relationships. J Appl Ecol. 2000; 37: 39–59.

[33] La Sorte F, Lee TM, Wilman H, Jetz W. Disparities between observed and predicted impacts of climate change on winter bird assemblages. Proc R Soc B. 2009; 276: 3167–3174. 10.1098/rspb.2009.0162 19520804 PMC2817117

[34] Seoane J, Carrascal LM. Interspecific differences in population trends of Spanish birds are related to habitat and climatic preferences. Global Ecol Biogeogr. 2008; 17: 111–121.

[35] Triviño M, Cabeza M, Thuiller W, Hickler T, Araújo MB. Risk assessment for Iberian birds under global change. Biol Conserv. 2013; 168: 192–200.

[36] Carrascal LM, Villén-Pérez S, Seoane J. Thermal, food and vegetation effects on winter bird species richness of Mediterranean oakwoods. Ecol Res. 2012; 27: 293–302.

[37] García-Ruiz JM, López-Moreno JI, Vicente-Serrano SM, Lasanta–Martínez T, Beguería S. Mediterranean water resources in a global change scenario. Earth-Sci Rev. 2011; 105: 121–139.

[38] Guo Q, Hu Z, Li S, Li X, Sun X, Yu G. Spatial variations in aboveground net primary productivity along a climate gradient in Eurasian temperate grassland: effects of mean annual precipitation and its seasonal distribution. Global Change Biol. 2012; 18: 3624–3631.

[39] Carrascal LM, Palomino D. Variación geográfica de la riqueza de especies invernantes en la península Ibérica. Estacionalidad y determinismo ambiental In: del Moral JC, Molina B, Bermejo A, Palomino D, Atlas de las Aves en Invierno en España 2007–2010. Madrid: Ministerio de Agricultura, Alimentación y Medio Ambiente-SEO/BirdLife; 2012 pp. 36–47.

[40] Tellería JL, Fandos G, López JF, Onrubia A, Refoyo P. Winter Distribution of Passerine Richness in the Maghreb (North Africa): A Conservation Assessment. Ardeola. 2014; 61: 335–350.

[41] Dunning JB. CRC handbook of avian body masses Boca Ratón: CRC Press; 1992.

[42] Cramp S. The birds of the Western Palearctic. Vol. V Oxford: Oxford University Press; 1988.

[43] Cramp S. The Birds of the Western Palearctic. Volume VI Oxford: Oxford University Press; 1992.

[44] Cuesta M, Balmori A. Mosquitero Común y Mosquitero Ibérico. In: Martí R, del Moral JC, Atlas de las aves reproductoras de España Madrid: Dirección General de Conservación de la Naturaleza-SEO pp: 489–491.

[45] Tellería JL, Asensio B, Díaz M. Aves Ibéricas Vol. II Paseriformes. Madrid: Reyero Publisher; 1999.

[46] Thévenot M, Vernon JDR, Bergier P. The Birds of Morocco. Tring: British Ornithologist Union; 2003

[47] Del Moral JC, Molina B, Bermejo A, Palomino D 2013 Atlas de las Aves en Invierno en España 2007–2010 Madrid: Ministerio de Agricultura, Alimentación y Medio Ambiente- SEO/BirdLife.

[48] De la Hera I, Pérez-Tris J, Tellería JL. Habitat distribution of migratory and sedentary blackcaps *Sylvia atricapilla* wintering in southern Iberia: a morphological and biogeochemical approach. J Avian Biol. 2012; 43: 333–340.

[49] Tellería JL, Fernández-López J, Fandos G. Using ring records and field surveys to predict the winter distribution of a migratory passerine. Bird Study. 2014; 61: 527–536.

[50] Levey D, Stiles FG. Evolutionary precursors of long-distance migration: resource availability and movement patterns in Neotropical landbirds. Am Nat. 1992; 140: 447–476.

[51] Bibby CJ, Burgess ND, Hill DA, Mustoe SH. Bird Census Techniques. London: Elsevier; 2000.

[52] Hijmans RJ, Cameron SE, Parra JL, Jones PG, Jarvis A. Very high resolution interpolated climate surfaces for global land areas. Int J Climatol. 2005; 25:1965–1978.

[53] Alerstam T, Enckell P. Unpredictable habitats and evolution of bird migration. Oikos. 1979; 33: 228–232.

[54] Wisz MS, Walther BA, Rahbek C. Using potential distributions to explore determinants of Western Palaearctic migratory songbird species richness in sub Saharan Africa. J Biogeogr. 2007; 34: 828–841.

[55] Legendre P. Spatial autocorrelation: trouble or new paradigm? Ecology. 1993; 74: 1659–1673.

[56] Jackman S. pscl: Classes and Methods for R Developed in the Political Science. R package version 1.4.9. 2015. Available: http://pscl.stanford.edu/

[57] Zuur A, Ieno EN, Walker N, Saveliev AA, Smith GM. Mixed effects models and extensions in ecology with R New York: Springer Science & Business Media; 2009.

[58] Burnham KP, Anderson DR. Model selection and multimodel inference: a practical information–theoretic approach New York: Springer; 2002.

[59] Zeileis A, Hothorn T. Diagnostic Checking in Regression Relationships. R News. 2002; 2(3), 7–10. Available: http://CRAN.R-project.org/doc/Rnews/

[60] Sanderson EW, Jaiteh M, Levy MA, Redford KH, Wannebo AV, Woolmer G. The human footprints and the last of the wild. BioScience. 2002; 52: 891–904.

[61] Stocker TF, Qin D, Plattner GK, Tignor M, Allen SK et al Fifth Assessment Report of the Intergovernmental Panel on Climate Change. Cambridge: Cambridge University Press; 2013

[62] Harris RMB, Grose MR, Lee G, Bindoff NL, Porfirio LL, Fox-Hughes P. Climate projections for ecologists. Clim Change. 2014; 5: 621–637.

[63] Grass Development Team. Geographic Resources Analysis Support System (GRASS) Software. Version 6.4.2. Open Source Geospatial Foundation, 2012 Available: http://grass.osgeo.org

[64] McGill BJ. Matters of scale. Science. 2010; 328: 575–576. 10.1126/science.1188528 20431001

[65] Zink RM. The evolution of avian migration. Biol J Linn Soc. 2011; 104: 237–250.

[66] Triviño M, Thuiller W, Cabeza M, Hickler T, Araújo MB. The contribution of vegetation and landscape configuration for predicting environmental change impacts on Iberian birds. Plos One, 2011; 6(12): e293732011 10.1371/journal.pone.0029373 PMC3245269 22216263

[67] Khaliq I, Hof C, Prinzinger R, Böhning-Gaese K, Pfenninger M. Global variation in thermal tolerances and vulnerability of endotherms to climate change. Proc R Soc B. 2014; 281: 20141097 10.1098/rspb.2014.1097 25009066 PMC4100521

[68] Swanson DL, Garland T. The evolution of high summit metabolism and cold tolerance in birds and its impact on present-day distributions. Evolution. 2008; 63: 184–194. 10.1111/j.1558-5646.2008.00522.x 18803689

[69] Root TL. Energy constraints on avian distributions and abundances. Ecology. 1988; 69: 330–339.

[70] Fernández- Alés R, Laffarga JM, Ortega F. Strategies in Mediterranean grassland annuals in relation to stress and disturbance. J Veg Sci. 1993; 4: 313–322.

[71] Doblas-Miranda E, Sánchez-Piñero F, González-Megías A. Vertical distribution of soil macrofauna in an arid ecosystem: Are litter and belowground compartmentalized habitats? Pedobiologia. 2009; 52: 361–373.

[72] IPCC. Synthesis Report. Contribution of Working Groups I, II and III to the Fifth Assessment Report of the Intergovernmental Panel on Climate Change, Geneva: Intergovernmental Panel on Climate Change, 2014

[73] Kotlaski S, Bosshard T, Lïthi D, Pall P, Schär C. Elevation gradients of European climate change in the regional climate model COSMO-CLM. Climatic Change. 2012; 112: 189–215.

[74] Rangwala I, Miller JR. Climate change in mountains: a review of elevation-dependent warming and its possible causes. Climatic Change. 2012; 114: 527–547.

[75] Clavero M, Villero D, Brotons L. Climate change or land use dynamics: Do we know what climate change indicators indicate? Plos One. 2011; 6: e18581 10.1371/journal.pone.0018581 21533025 PMC3080866

[76] Hawkins BA, Porter EE, Diniz-Filho JAF. Productivity and history as predictors of the latitudinal diversity gradient of terrestrial birds. Ecology. 2003; 84: 1608–1623

[77] Pearce‐Higgins JW, Eglington SM, Martay B, Chamberlain DE. Drivers of climate change impacts on bird communities. J Anim Ecol. 2015; 84:943–954. 10.1111/1365-2656.12364 25757576

[78] La Sorte F, Butchart SHM, Jetz W, Böhning-Gaese K. Range-wide latitudinal and elevational temperature gradients for the World´s Terrestrial Birds: Implications under Global Climate Change. Plos One. 2012; 9: e98361 10.1371/journal.pone.0098361 PMC4031198 24852009

[79] Hampe A, Petit RJ. Conserving biodiversity under climate change: the rear edge matters. Ecol Lett. 2005; 8: 461–467. 10.1111/j.1461-0248.2005.00739.x 21352449

